# Dibromido(2,2′-dimethyl-4,4′-bi-1,3-thia­zole-κ^2^ *N*,*N*′)mercury(II)

**DOI:** 10.1107/S1600536810051494

**Published:** 2010-12-15

**Authors:** Anita Abedi

**Affiliations:** a Department of Chemistry, Islamic Azad University, North Tehran Branch, Tehran, Iran

## Abstract

The asymmetric unit of the title compound, [HgBr_2_(C_8_H_8_N_2_S_2_)], contains two crystallographically independent mol­ecules. The Hg^II^ atoms are four-coordinated in a distorted tetra­hedral geometry by two N atoms from a 2,2′-dimethyl-4,4′-bi-1,3-thia­zole ligand and two Br atoms. In the crystal structure, inter­molecular C—H⋯Br hydrogen bonds and π–π contacts between the thia­zole rings [centroid–centroid distances = 3.670 (3) and 3.614 (2) Å] stabilize the structure.

## Related literature

For metal complexes with 2,2′-dimethyl-4,4′-bithia­zole ligands, see: Abedi & Yahyazade Bali (2010 ▶); Al-Hashemi *et al.* (2009 ▶, 2010 ▶); Khavasi *et al.* (2008 ▶); Notash *et al.* (2008 ▶, 2009 ▶). For related structures, see: Kalateh *et al.* (2008 ▶); Safari *et al.* (2009 ▶). 
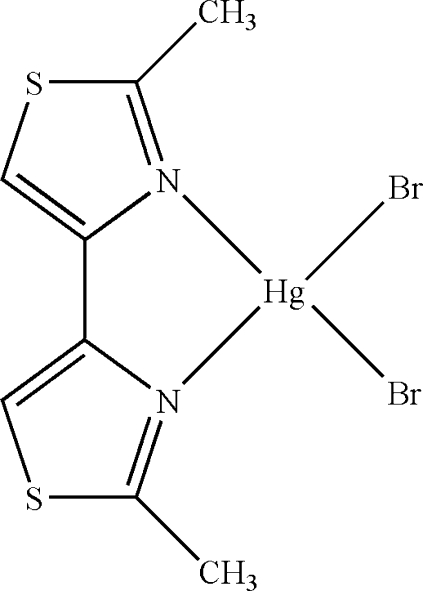


## Experimental

### 

#### Crystal data

[HgBr_2_(C_8_H_8_N_2_S_2_)]
*M* *_r_* = 556.69
Triclinic, 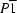

*a* = 10.2799 (6) Å
*b* = 11.1595 (7) Å
*c* = 11.6821 (7) Å
α = 88.4456 (11)°
β = 85.3290 (11)°
γ = 77.1162 (11)°
*V* = 1302.02 (14) Å^3^
*Z* = 4
Mo *K*α radiation
μ = 18.25 mm^−1^
*T* = 100 K
0.14 × 0.12 × 0.08 mm

#### Data collection

Bruker APEXII CCD diffractometer
Absorption correction: multi-scan (*SADABS*; Bruker, 2001 ▶) *T*_min_ = 0.070, *T*_max_ = 0.240
20881 measured reflections
6912 independent reflections
5865 reflections with *I* > 2σ(*I*)
*R*_int_ = 0.041

#### Refinement

*R*[*F* ^2^ > 2σ(*F* ^2^)] = 0.026
*wR*(*F* ^2^) = 0.062
*S* = 0.94
6912 reflections
275 parameters
H-atom parameters constrained
Δρ_max_ = 1.28 e Å^−3^
Δρ_min_ = −1.68 e Å^−3^

### 

Data collection: *APEX2* (Bruker, 2007 ▶); cell refinement: *SAINT* (Bruker, 2007 ▶); data reduction: *SAINT*; program(s) used to solve structure: *SHELXS97* (Sheldrick, 2008 ▶); program(s) used to refine structure: *SHELXL97* (Sheldrick, 2008 ▶); molecular graphics: *SHELXTL* (Sheldrick, 2008 ▶) and *Mercury* (Macrae *et al.*, 2006 ▶); software used to prepare material for publication: *SHELXTL*.

## Supplementary Material

Crystal structure: contains datablocks I, global. DOI: 10.1107/S1600536810051494/hy2386sup1.cif

Structure factors: contains datablocks I. DOI: 10.1107/S1600536810051494/hy2386Isup2.hkl

Additional supplementary materials:  crystallographic information; 3D view; checkCIF report

## Figures and Tables

**Table 1:** Selected bond lengths (Å)

Hg1—N1	2.379 (4)
Hg1—N2	2.383 (4)
Hg1—Br1	2.4970 (5)
Hg1—Br2	2.5206 (5)
Hg2—N3	2.357 (4)
Hg2—N4	2.410 (4)
Hg2—Br3	2.4999 (5)
Hg2—Br4	2.4957 (5)

**Table 2:** Hydrogen-bond geometry (Å, °)

*D*—H⋯*A*	*D*—H	H⋯*A*	*D*⋯*A*	*D*—H⋯*A*
C8—H8*B*⋯Br4^i^	0.98	2.92	3.826 (5)	155
C10—H10*A*⋯Br4^i^	0.95	2.92	3.760 (5)	148
C16—H16*B*⋯Br3^ii^	0.98	2.87	3.772 (5)	154
C16—H16*C*⋯Br2^iii^	0.98	2.88	3.837 (5)	165

Symmetry codes: (i) 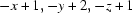; (ii) 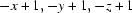; (iii) 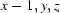.

**Fig. 1.**
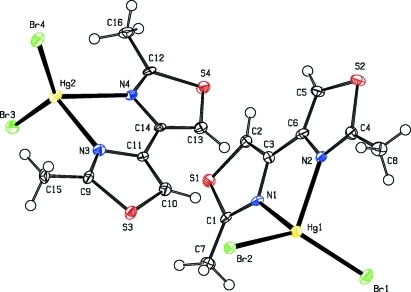
The molecular structure of the title compound. Displacement ellipsoids are drawn at the 50% probability level.

**Fig. 2.**
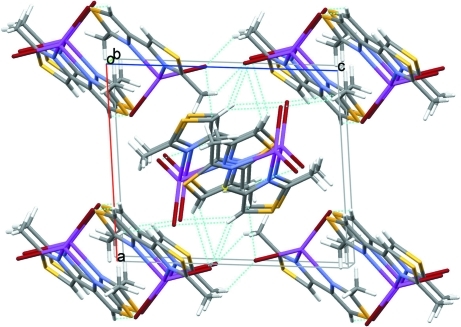
Crystal packing diagram for the title compound. Dashed lines denote hydrogen bonds.

## supplementary crystallographic information

### Comment

Recently, we reported the synthesis and crystal structure of [HgI_2_(dm4bt)] (dm4bt is 2,2'-dimethyl-4,4'-bithiazole) (Abedi & Yahyazade Bali, 2010). Dm4bt is a good bidentate ligand, and numerous complexes with dm4bt have been prepared, such as that of zinc (Khavasi *et al.*, 2008), thallium (Notash *et al.*, 2008), cadmium (Notash *et al.*, 2009) and copper (Al-Hashemi *et al.*, 2009, 2010). For further investigation of dm4bt, we synthesis the title complex and report herein its crystal structure.

The asymmetric unit of the title compound (Fig. 1) contains two crystallographically independent molecules. The Hg^II^ atom is four-coordinated in a distorted tetrahedral configuration by two N atoms from a dm4bt ligand and two Br atoms. The Hg—N and Hg—Br bond lengths (Table 1) and angles are within normal range found in [Hg(SCN)_2_(dm4bt)] (Safari *et al.*, 2009) and {HgBr_2_[NH(py)_2_]} [NH(py)_2_ is di-2-pyridylamine] (Kalateh *et al.*, 2008).

In the crystal structure, intermolecular C—H···Br hydrogen bonds (Table 2) and π–π contacts (Fig. 2) between the thiazole rings, *Cg*1···*Cg*2^i^ and *Cg*3···*Cg*4^ii^ [symmetry codes: (i) 2-x, 1-y, -z; (ii) 1-x, 2-y, 1-z. *Cg*1, *Cg*2, *Cg*3 and *Cg*4 are the centroids of the rings S1/C1/N1/C3/C2, S2/C4/N2/C6/C5, S3/C9/N3/C11/C10 and S4/C12/N4/C14/C13, respectively] stabilize the structure, with centroid–centroid distances of 3.670 (3) and 3.614 (2) Å.

### Experimental

For the preparation of the title compound, a solution of dm4bt (0.26 g, 1.3 mmol) in methanol (15 ml) was added to a solution of HgBr_2_ (0.47 g, 1.3 mmol) in methanol (15 ml) at room temperature. Crystals suitable for X-ray diffraction experiment were obtained by methanol diffusion into a colorless solution in DMSO after one week (yield: 0.52 g, 71.8%).

### Refinement

H atoms were positioned geometrically and refined as riding atoms, with C—H = 0.95 (CH) and 0.98 (CH_3_) Å and with *U*_iso_(H) = 1.2(1.5 for methyl)*U*_eq_(C). The highest residual electron density was found at 0.85 Å from Hg2 atom and the deepest hole at 0.70 Å from Hg1 atom.

### Crystal data

**Table:**

[HgBr_2_(C_8_H_8_N_2_S_2_)]	*Z* = 4
*M**_r_* = 556.69	*F*(000) = 1008
Triclinic, *P*1	*D*_x_ = 2.840 Mg m^−^^3^
Hall symbol: -P 1	Mo *K*α radiation, λ = 0.71073 Å
*a* = 10.2799 (6) Å	Cell parameters from 2931 reflections
*b* = 11.1595 (7) Å	θ = 3.0–29.0°
*c* = 11.6821 (7) Å	µ = 18.25 mm^−^^1^
α = 88.4456 (11)°	*T* = 100 K
β = 85.3290 (11)°	Prism, colorless
γ = 77.1162 (11)°	0.14 × 0.12 × 0.08 mm
*V* = 1302.02 (14) Å^3^	

### Data collection

**Table:**

Bruker APEXII CCD diffractometer	6912 independent reflections
Radiation source: fine-focus sealed tube	5865 reflections with *I* > 2σ(*I*)
graphite	*R*_int_ = 0.041
φ and ω scans	θ_max_ = 29.0°, θ_min_ = 1.8°
Absorption correction: multi-scan (*SADABS*; Bruker, 2001)	*h* = −14→14
*T*_min_ = 0.070, *T*_max_ = 0.240	*k* = −15→15
20881 measured reflections	*l* = −15→15

### Refinement

**Table:**

Refinement on *F*^2^	Primary atom site location: structure-invariant direct methods
Least-squares matrix: full	Secondary atom site location: difference Fourier map
*R*[*F*^2^ > 2σ(*F*^2^)] = 0.026	Hydrogen site location: inferred from neighbouring sites
*wR*(*F*^2^) = 0.062	H-atom parameters constrained
*S* = 0.94	*w* = 1/[σ^2^(*F*_o_^2^) + (0.0299*P*)^2^ + 2.0838*P*] where *P* = (*F*_o_^2^ + 2*F*_c_^2^)/3
6912 reflections	(Δ/σ)_max_ = 0.002
275 parameters	Δρ_max_ = 1.28 e Å^−^^3^
0 restraints	Δρ_min_ = −1.68 e Å^−^^3^

### Fractional atomic coordinates and isotropic or equivalent isotropic displacement parameters (Å^2^)

**Table:**

	*x*	*y*	*z*	*U*_iso_*/*U*_eq_	
Hg1	1.080846 (18)	0.703396 (17)	0.207265 (14)	0.01438 (5)	
Br1	1.27509 (5)	0.73077 (5)	0.07429 (4)	0.01974 (10)	
Br2	1.01869 (5)	0.79183 (4)	0.40562 (4)	0.01472 (9)	
S1	0.96732 (13)	0.30682 (11)	0.30191 (10)	0.0162 (2)	
S2	0.74266 (12)	0.71656 (11)	−0.06846 (10)	0.0167 (2)	
N1	1.0123 (4)	0.5145 (4)	0.2400 (3)	0.0126 (8)	
N2	0.9123 (4)	0.6940 (3)	0.0815 (3)	0.0118 (7)	
C1	1.0489 (4)	0.4251 (4)	0.3135 (4)	0.0117 (8)	
C2	0.8814 (5)	0.3826 (4)	0.1925 (4)	0.0160 (9)	
H2A	0.8189	0.3523	0.1522	0.019*	
C3	0.9157 (5)	0.4932 (4)	0.1714 (4)	0.0129 (9)	
C4	0.8605 (5)	0.7689 (4)	0.0020 (4)	0.0148 (9)	
C5	0.7668 (5)	0.5861 (4)	0.0144 (4)	0.0150 (9)	
H5A	0.7206	0.5217	0.0097	0.018*	
C6	0.8618 (5)	0.5883 (4)	0.0881 (4)	0.0131 (9)	
C7	1.1512 (5)	0.4226 (5)	0.3961 (4)	0.0186 (10)	
H7A	1.1390	0.5043	0.4296	0.028*	
H7B	1.2407	0.3995	0.3561	0.028*	
H7C	1.1418	0.3625	0.4571	0.028*	
C8	0.8973 (5)	0.8888 (4)	−0.0288 (4)	0.0189 (10)	
H8A	0.9945	0.8786	−0.0294	0.028*	
H8B	0.8531	0.9507	0.0280	0.028*	
H8C	0.8684	0.9155	−0.1050	0.028*	
Hg2	0.411665 (18)	0.785100 (16)	0.717555 (15)	0.01467 (5)	
Br3	0.50713 (5)	0.58257 (4)	0.80847 (4)	0.01660 (10)	
Br4	0.19137 (5)	0.93379 (4)	0.74712 (4)	0.01734 (10)	
S3	0.72536 (12)	1.05628 (11)	0.68030 (10)	0.0150 (2)	
S4	0.59092 (12)	0.68616 (11)	0.31954 (10)	0.0161 (2)	
N3	0.5664 (4)	0.9109 (4)	0.6782 (3)	0.0142 (8)	
N4	0.5044 (4)	0.7580 (3)	0.5213 (3)	0.0110 (7)	
C9	0.6015 (5)	0.9912 (4)	0.7442 (4)	0.0136 (9)	
C10	0.7285 (5)	0.9747 (4)	0.5581 (4)	0.0154 (9)	
H10A	0.7849	0.9794	0.4904	0.018*	
C11	0.6378 (4)	0.9014 (4)	0.5721 (4)	0.0114 (8)	
C12	0.4840 (4)	0.6864 (4)	0.4407 (4)	0.0138 (9)	
C13	0.6630 (5)	0.7909 (4)	0.3806 (4)	0.0137 (9)	
H13A	0.7328	0.8249	0.3443	0.016*	
C14	0.6059 (4)	0.8182 (4)	0.4879 (4)	0.0100 (8)	
C15	0.5358 (5)	1.0239 (5)	0.8604 (4)	0.0176 (10)	
H15A	0.4717	0.9721	0.8807	0.026*	
H15B	0.4885	1.1105	0.8608	0.026*	
H15C	0.6036	1.0107	0.9164	0.026*	
C16	0.3766 (5)	0.6164 (5)	0.4523 (4)	0.0181 (10)	
H16A	0.3632	0.5912	0.5326	0.027*	
H16B	0.4023	0.5434	0.4038	0.027*	
H16C	0.2932	0.6685	0.4281	0.027*	

### Atomic displacement parameters (Å^2^)

**Table:**

	*U*^11^	*U*^22^	*U*^33^	*U*^12^	*U*^13^	*U*^23^
Hg1	0.01399 (9)	0.01808 (10)	0.01340 (8)	−0.00844 (7)	−0.00148 (6)	0.00061 (6)
Br1	0.0193 (2)	0.0236 (3)	0.0190 (2)	−0.0124 (2)	0.00513 (19)	−0.00411 (18)
Br2	0.0117 (2)	0.0184 (2)	0.0138 (2)	−0.00327 (17)	0.00036 (16)	−0.00020 (17)
S1	0.0190 (6)	0.0165 (6)	0.0161 (5)	−0.0099 (5)	−0.0037 (4)	0.0045 (4)
S2	0.0160 (6)	0.0177 (6)	0.0179 (5)	−0.0051 (5)	−0.0079 (5)	0.0019 (4)
N1	0.0086 (18)	0.015 (2)	0.0150 (18)	−0.0052 (15)	−0.0010 (15)	0.0000 (15)
N2	0.0097 (18)	0.0125 (19)	0.0134 (18)	−0.0036 (15)	0.0000 (14)	0.0017 (14)
C1	0.007 (2)	0.012 (2)	0.015 (2)	−0.0021 (16)	0.0031 (16)	−0.0011 (16)
C2	0.017 (2)	0.020 (2)	0.014 (2)	−0.011 (2)	−0.0026 (18)	0.0032 (18)
C3	0.013 (2)	0.015 (2)	0.011 (2)	−0.0035 (18)	−0.0011 (17)	0.0001 (16)
C4	0.017 (2)	0.018 (2)	0.011 (2)	−0.0052 (19)	−0.0008 (17)	−0.0031 (17)
C5	0.014 (2)	0.016 (2)	0.015 (2)	−0.0045 (18)	−0.0055 (18)	0.0006 (17)
C6	0.009 (2)	0.016 (2)	0.015 (2)	−0.0045 (18)	0.0010 (17)	−0.0052 (17)
C7	0.018 (2)	0.022 (3)	0.019 (2)	−0.008 (2)	−0.0075 (19)	0.0053 (19)
C8	0.020 (3)	0.015 (2)	0.023 (2)	−0.005 (2)	−0.004 (2)	−0.0003 (19)
Hg2	0.01185 (9)	0.01587 (9)	0.01664 (9)	−0.00476 (7)	0.00171 (7)	−0.00051 (6)
Br3	0.0161 (2)	0.0170 (2)	0.0169 (2)	−0.00399 (18)	−0.00171 (17)	0.00080 (17)
Br4	0.0137 (2)	0.0168 (2)	0.0201 (2)	−0.00250 (18)	0.00373 (18)	0.00131 (17)
S3	0.0160 (6)	0.0169 (6)	0.0139 (5)	−0.0084 (5)	0.0024 (4)	−0.0025 (4)
S4	0.0136 (5)	0.0197 (6)	0.0151 (5)	−0.0042 (5)	0.0012 (4)	−0.0057 (4)
N3	0.0134 (19)	0.015 (2)	0.0149 (18)	−0.0048 (16)	−0.0012 (15)	−0.0004 (15)
N4	0.0065 (17)	0.0114 (18)	0.0140 (17)	0.0002 (14)	0.0005 (14)	−0.0018 (14)
C9	0.012 (2)	0.015 (2)	0.013 (2)	−0.0031 (17)	0.0027 (17)	0.0006 (17)
C10	0.015 (2)	0.017 (2)	0.014 (2)	−0.0043 (18)	0.0002 (18)	−0.0004 (17)
C11	0.007 (2)	0.015 (2)	0.012 (2)	−0.0022 (17)	−0.0010 (16)	0.0004 (16)
C12	0.0029 (19)	0.020 (2)	0.017 (2)	0.0006 (17)	0.0003 (16)	−0.0006 (18)
C13	0.011 (2)	0.013 (2)	0.017 (2)	−0.0019 (17)	−0.0004 (17)	−0.0004 (17)
C14	0.0051 (19)	0.009 (2)	0.015 (2)	−0.0003 (16)	−0.0022 (16)	0.0033 (16)
C15	0.022 (3)	0.023 (3)	0.009 (2)	−0.009 (2)	0.0028 (18)	−0.0022 (18)
C16	0.012 (2)	0.023 (3)	0.021 (2)	−0.0072 (19)	−0.0017 (19)	−0.006 (2)

### Geometric parameters (Å, °)

**Table:**

Hg1—N1	2.379 (4)	Hg2—N3	2.357 (4)
Hg1—N2	2.383 (4)	Hg2—N4	2.410 (4)
Hg1—Br1	2.4970 (5)	Hg2—Br3	2.4999 (5)
Hg1—Br2	2.5206 (5)	Hg2—Br4	2.4957 (5)
S1—C2	1.709 (5)	S3—C10	1.708 (5)
S1—C1	1.727 (4)	S3—C9	1.713 (5)
S2—C5	1.708 (5)	S4—C13	1.715 (5)
S2—C4	1.729 (5)	S4—C12	1.720 (5)
N1—C1	1.309 (6)	N3—C9	1.324 (6)
N1—C3	1.389 (6)	N3—C11	1.383 (6)
N2—C4	1.298 (6)	N4—C12	1.310 (6)
N2—C6	1.388 (6)	N4—C14	1.387 (5)
C1—C7	1.480 (6)	C9—C15	1.483 (6)
C2—C3	1.368 (6)	C10—C11	1.368 (6)
C2—H2A	0.9500	C10—H10A	0.9500
C3—C6	1.467 (7)	C11—C14	1.475 (6)
C4—C8	1.496 (7)	C12—C16	1.483 (6)
C5—C6	1.358 (6)	C13—C14	1.353 (6)
C5—H5A	0.9500	C13—H13A	0.9500
C7—H7A	0.9800	C15—H15A	0.9800
C7—H7B	0.9800	C15—H15B	0.9800
C7—H7C	0.9800	C15—H15C	0.9800
C8—H8A	0.9800	C16—H16A	0.9800
C8—H8B	0.9800	C16—H16B	0.9800
C8—H8C	0.9800	C16—H16C	0.9800
			
N1—Hg1—N2	71.23 (13)	N3—Hg2—N4	70.25 (13)
N1—Hg1—Br1	124.79 (9)	N3—Hg2—Br4	104.07 (10)
N2—Hg1—Br1	103.63 (9)	N4—Hg2—Br4	115.64 (9)
N1—Hg1—Br2	98.36 (9)	N3—Hg2—Br3	114.30 (10)
N2—Hg1—Br2	120.67 (9)	N4—Hg2—Br3	102.77 (9)
Br1—Hg1—Br2	126.492 (16)	Br4—Hg2—Br3	132.848 (17)
C2—S1—C1	90.8 (2)	C10—S3—C9	90.6 (2)
C5—S2—C4	90.4 (2)	C13—S4—C12	90.4 (2)
C1—N1—C3	112.7 (4)	C9—N3—C11	111.5 (4)
C1—N1—Hg1	131.2 (3)	C9—N3—Hg2	130.2 (3)
C3—N1—Hg1	116.2 (3)	C11—N3—Hg2	118.3 (3)
C4—N2—C6	111.8 (4)	C12—N4—C14	112.3 (4)
C4—N2—Hg1	131.6 (3)	C12—N4—Hg2	130.9 (3)
C6—N2—Hg1	116.5 (3)	C14—N4—Hg2	116.6 (3)
N1—C1—C7	124.3 (4)	N3—C9—C15	122.7 (4)
N1—C1—S1	112.7 (3)	N3—C9—S3	113.5 (3)
C7—C1—S1	123.0 (4)	C15—C9—S3	123.8 (4)
C3—C2—S1	110.1 (3)	C11—C10—S3	110.1 (3)
C3—C2—H2A	125.0	C11—C10—H10A	125.0
S1—C2—H2A	125.0	S3—C10—H10A	125.0
C2—C3—N1	113.8 (4)	C10—C11—N3	114.4 (4)
C2—C3—C6	127.8 (4)	C10—C11—C14	128.1 (4)
N1—C3—C6	118.4 (4)	N3—C11—C14	117.5 (4)
N2—C4—C8	124.7 (4)	N4—C12—C16	123.1 (4)
N2—C4—S2	113.3 (3)	N4—C12—S4	112.9 (3)
C8—C4—S2	122.0 (4)	C16—C12—S4	124.0 (4)
C6—C5—S2	109.8 (4)	C14—C13—S4	110.1 (3)
C6—C5—H5A	125.1	C14—C13—H13A	124.9
S2—C5—H5A	125.1	S4—C13—H13A	124.9
C5—C6—N2	114.7 (4)	C13—C14—N4	114.2 (4)
C5—C6—C3	127.7 (4)	C13—C14—C11	128.8 (4)
N2—C6—C3	117.7 (4)	N4—C14—C11	117.0 (4)
C1—C7—H7A	109.5	C9—C15—H15A	109.5
C1—C7—H7B	109.5	C9—C15—H15B	109.5
H7A—C7—H7B	109.5	H15A—C15—H15B	109.5
C1—C7—H7C	109.5	C9—C15—H15C	109.5
H7A—C7—H7C	109.5	H15A—C15—H15C	109.5
H7B—C7—H7C	109.5	H15B—C15—H15C	109.5
C4—C8—H8A	109.5	C12—C16—H16A	109.5
C4—C8—H8B	109.5	C12—C16—H16B	109.5
H8A—C8—H8B	109.5	H16A—C16—H16B	109.5
C4—C8—H8C	109.5	C12—C16—H16C	109.5
H8A—C8—H8C	109.5	H16A—C16—H16C	109.5
H8B—C8—H8C	109.5	H16B—C16—H16C	109.5
			
N2—Hg1—N1—C1	−178.0 (4)	N4—Hg2—N3—C9	177.3 (5)
Br1—Hg1—N1—C1	88.2 (4)	Br4—Hg2—N3—C9	64.7 (4)
Br2—Hg1—N1—C1	−58.3 (4)	Br3—Hg2—N3—C9	−87.2 (4)
N2—Hg1—N1—C3	1.3 (3)	N4—Hg2—N3—C11	−4.7 (3)
Br1—Hg1—N1—C3	−92.5 (3)	Br4—Hg2—N3—C11	−117.3 (3)
Br2—Hg1—N1—C3	121.0 (3)	Br3—Hg2—N3—C11	90.8 (3)
N1—Hg1—N2—C4	−177.5 (4)	N3—Hg2—N4—C12	178.4 (4)
Br1—Hg1—N2—C4	−55.0 (4)	Br4—Hg2—N4—C12	−85.0 (4)
Br2—Hg1—N2—C4	93.8 (4)	Br3—Hg2—N4—C12	66.9 (4)
N1—Hg1—N2—C6	−0.9 (3)	N3—Hg2—N4—C14	5.4 (3)
Br1—Hg1—N2—C6	121.7 (3)	Br4—Hg2—N4—C14	101.9 (3)
Br2—Hg1—N2—C6	−89.5 (3)	Br3—Hg2—N4—C14	−106.2 (3)
C3—N1—C1—C7	179.2 (4)	C11—N3—C9—C15	176.8 (4)
Hg1—N1—C1—C7	−1.5 (7)	Hg2—N3—C9—C15	−5.0 (7)
C3—N1—C1—S1	0.6 (5)	C11—N3—C9—S3	−1.2 (5)
Hg1—N1—C1—S1	179.9 (2)	Hg2—N3—C9—S3	177.0 (2)
C2—S1—C1—N1	0.3 (4)	C10—S3—C9—N3	1.2 (4)
C2—S1—C1—C7	−178.3 (4)	C10—S3—C9—C15	−176.8 (5)
C1—S1—C2—C3	−1.2 (4)	C9—S3—C10—C11	−0.9 (4)
S1—C2—C3—N1	1.7 (5)	S3—C10—C11—N3	0.4 (5)
S1—C2—C3—C6	−177.4 (4)	S3—C10—C11—C14	178.7 (4)
C1—N1—C3—C2	−1.5 (6)	C9—N3—C11—C10	0.5 (6)
Hg1—N1—C3—C2	179.0 (3)	Hg2—N3—C11—C10	−177.9 (3)
C1—N1—C3—C6	177.7 (4)	C9—N3—C11—C14	−178.0 (4)
Hg1—N1—C3—C6	−1.7 (5)	Hg2—N3—C11—C14	3.6 (5)
C6—N2—C4—C8	−178.1 (4)	C14—N4—C12—C16	−176.9 (4)
Hg1—N2—C4—C8	−1.3 (7)	Hg2—N4—C12—C16	9.8 (7)
C6—N2—C4—S2	1.5 (5)	C14—N4—C12—S4	1.2 (5)
Hg1—N2—C4—S2	178.2 (2)	Hg2—N4—C12—S4	−172.1 (2)
C5—S2—C4—N2	−0.4 (4)	C13—S4—C12—N4	−1.5 (4)
C5—S2—C4—C8	179.2 (4)	C13—S4—C12—C16	176.6 (4)
C4—S2—C5—C6	−0.8 (4)	C12—S4—C13—C14	1.3 (4)
S2—C5—C6—N2	1.9 (5)	S4—C13—C14—N4	−0.9 (5)
S2—C5—C6—C3	−177.9 (4)	S4—C13—C14—C11	178.8 (4)
C4—N2—C6—C5	−2.2 (6)	C12—N4—C14—C13	−0.2 (6)
Hg1—N2—C6—C5	−179.5 (3)	Hg2—N4—C14—C13	174.1 (3)
C4—N2—C6—C3	177.6 (4)	C12—N4—C14—C11	−179.9 (4)
Hg1—N2—C6—C3	0.3 (5)	Hg2—N4—C14—C11	−5.6 (5)
C2—C3—C6—C5	−0.1 (8)	C10—C11—C14—C13	3.5 (8)
N1—C3—C6—C5	−179.3 (4)	N3—C11—C14—C13	−178.2 (5)
C2—C3—C6—N2	−179.9 (4)	C10—C11—C14—N4	−176.8 (5)
N1—C3—C6—N2	1.0 (6)	N3—C11—C14—N4	1.5 (6)

### Hydrogen-bond geometry (Å, °)

**Table:**

*D*—H···*A*	*D*—H	H···*A*	*D*···*A*	*D*—H···*A*
C8—H8B···Br4^i^	0.98	2.92	3.826 (5)	155
C10—H10A···Br4^i^	0.95	2.92	3.760 (5)	148
C16—H16B···Br3^ii^	0.98	2.87	3.772 (5)	154
C16—H16C···Br2^iii^	0.98	2.88	3.837 (5)	165

Symmetry codes: (i) −*x*+1, −*y*+2, −*z*+1; (ii) −*x*+1, −*y*+1, −*z*+1; (iii) *x*−1, *y*, *z*.

## References

[bb1] Abedi, A. & Yahyazade Bali, E. (2010). *Acta Cryst.* E**66**, m1023. 10.1107/S1600536810029302 PMC3007489 21588098

[bb2] Al-Hashemi, R., Safari, N., Abedi, A., Notash, B., Amani, V. & Khavasi, H. R. (2009). *J. Coord. Chem.* **62**, 2909–2918.

[bb3] Al-Hashemi, R., Safari, N., Amani, S., Amani, V., Abedi, A., Khavasi, H. R. & Ng, S. W. (2010). *J. Coord. Chem.* **63**, 3207–3217.

[bb4] Bruker (2001). *SADABS* Bruker AXS Inc., Madison, Wisconsin, USA.

[bb5] Bruker (2007). *APEX2* and *SAINT* Bruker AXS Inc., Madison, Wisconsin, USA.

[bb6] Kalateh, K., Norouzi, A., Ebadi, A., Ahmadi, R. & Amani, V. (2008). *Acta Cryst.* E**64**, m1583–m1584. 10.1107/S1600536808038129 PMC2960074 21581184

[bb7] Khavasi, H. R., Abedi, A., Amani, V., Notash, B. & Safari, N. (2008). *Polyhedron*, **27**, 1848–1854.

[bb8] Macrae, C. F., Edgington, P. R., McCabe, P., Pidcock, E., Shields, G. P., Taylor, R., Towler, M. & van de Streek, J. (2006). *J. Appl. Cryst.* **39**, 453–457.

[bb9] Notash, B., Safari, N., Abedi, A., Amani, V. & Khavasi, H. R. (2009). *J. Coord. Chem.* **62**, 1638–1649.

[bb10] Notash, B., Safari, N., Khavasi, H. R., Amani, V. & Abedi, A. (2008). *J. Organomet. Chem.* **693**, 3553–3557.

[bb11] Safari, N., Amani, V., Abedi, A., Notash, B. & Ng, S. W. (2009). *Acta Cryst.* E**65**, m372. 10.1107/S1600536809006904 PMC2969049 21582326

[bb12] Sheldrick, G. M. (2008). *Acta Cryst.* A**64**, 112–122. 10.1107/S0108767307043930 18156677

